# Melanopsin enhances image persistence

**DOI:** 10.1016/j.cub.2023.10.039

**Published:** 2023-11-14

**Authors:** Tom Woelders, Annette E. Allen, Robert J. Lucas

**Affiliations:** 1 Division of Neuroscience and Centre for Biological Timing, School of Biology, Faculty of Biology Medicine and Health, University of Manchester, Upper Brook Street, M13 9PT Manchester, UK

## Abstract

Contributions of the inner retinal photopigment melanopsin to human visual perception are incompletely understood. Here, we use a four-primary display to produce stimuli differing in melanopsin versus cone contrast in psychophysical paradigms in eight subjects with normal color vision. We address two predictions from electrophysiological recordings of the melanopsin system in non-human mammals: melanopsin influences color and/or supports image persistence under visual fixation. We first construct chromatic contrast sensi-tivity contours for stimuli differing in melanopsin excitation presented as a central annulus (10°) or peripheral (22.5°) spot. We find that although including melanopsin contrast produces modest changes in the average chromatic coordinates in both eccentricities, this occurs equally at low (0.5 Hz) and higher (3.75 Hz) temporal frequencies, arguing that it reflects divergence in cone spectral sensitivity in our participants from that captured in standardized cone fundamentals rather than a melanopsin contribution to color. We continue to ask whether the established ability of melanopsin to sustain firing of visual neurons under extended light exposure has a visual correlate, using the optical illusion of Troxler fading in which blurred spots in periphery disappear during visual fixation. We find that introducing additional melanopsin contrast (+28% Michelson contrast) to either bright or dark spots increases fading latency by 35% ± 8.8% and 41% ± 13.6%, respectively. Our data argue that the primary contribution of melanopsin to perception under these conditions is not to provide a color percept but rather to enhance persistence of low spatial frequency patterns during visual fixation.

## Introduction

Alongside the outer retinal rod and cone photoreceptors, the mammalian retina expresses a third class of photopigment, known as melanopsin, in a subclass of retinal ganglion cells, rendering these cells intrinsically photosensitive. These intrinsically photosensitive retinal ganglion cells (ipRGCs) integrate extrinsic rod and cone input with the intrinsic melanopsin-mediated response to light to send a composite signal to downstream brain areas.^1,2^ Melanopsin contributes to subconscious responses to ambient light such as circadian entrainment and the pupillary light reflex.^3–6^ However, across mammalian species, ipRGCs also target the lateral geniculate nucleus (LGN) of the thalamus,^7^ the relay station for input to the visual cortex and for visual perception.

Efforts to understand the significance of the melanopsin contribution to thalamocortical vision for human perception are complicated by the paucity of methods to selectively modulate melanopsin activity. One approach has been to adopt the method of silent substitution,^8^ in which the differential response between two spectra matched for cone, but not for melanopsin excitation (i.e., metamers), is used to assess the contribution of melanopsin to visually evoked responses to light. Such metameric stimuli have been shown to have different effectiveness for eliciting pupillary constriction^9,10^ and other unconscious light responses.^11–13^ Several studies have reported the impact of such selective modulation in melanopsin activation on aspects of human conscious vision.^14–19^ The most widely reported percept induced by stimuli providing selective modulation of melanopsin is a change in apparent brightness.^15,17–19^ This fits also with experiments in mice in which melanopsin knockout can be employed as an additional control.^18^

As melanopsin is far from the only photoreceptive mechanism responsible for brightness discrimination, an important question is under what circumstances a melanopsin brightness percept is a useful addition to that provided by cones (luminance). This question has been addressed most extensively in electrophysiological recordings of ipRGCs and their central targets in non-human mammals. The most reliable distinction between the contribution of melanopsin versus rod/cone signals to the activity of these neurons is revealed by a light step lasting several seconds. During the plateau portion of these light steps, maintained excitation relies disproportionately on melanopsin.^18,20–23^ The significance of this property has not been directly assessed for human vision. In human vision, stabilized images fade into the background (a phenomenon known as Troxler fading), indicating that just as the amplitude of electrophysiological responses mediated by cones dissipates under continuous illumination, so too does perception. It follows that one prediction of the contribution of melanopsin to electrophysiological activity is that it could contribute to counteracting Troxler fading. Here, we set out to test this prediction.

A second feature of the electrophysiological data, which has a straightforward prediction for perception, is the report that ipRGCs across species can receive cone opponent input. In macaque, this corresponds to an L + M-on/S-off arrangement,^1^ which (as melanopsin is always excitatory) raises the possibility of a melanopsin contribution to an L + M-on/S-off chromatic axis. The psychophysical literature on this topic is currently contradictory. Several studies have explored the color appearance of metamers differing in melanopsin excitation, with some inferring a melanopic contribution to color,^16,24^ some reporting color differences without attributing them to melanopsin,^25^ and others not finding chromatic differences at all.^18^ More systematic analyses have also produced contradictory outcomes. A report that including melanopsin contrast does not impact thresholds for discrimination in various color directions^18^ suggests that melanopsin provides no color percept. Conversely, color-matching paradigms have defined melanopsin contrast as perceptually equivalent to a decrease in the S/(L + M) direction.^16,24^ In an attempt to resolve this inconsistency, we set out to undertake a color-matching approach with the additional control of modulating temporal frequency. Melanopsin reliably responds best to lower temporal frequencies;^16,20,26,27^ we therefore expect any melanopsin contribution to color to be more apparent at lower versus higher temporal frequencies.

## Results

### Melanopsin contrast does not impact color-matching

**Figure 1:**
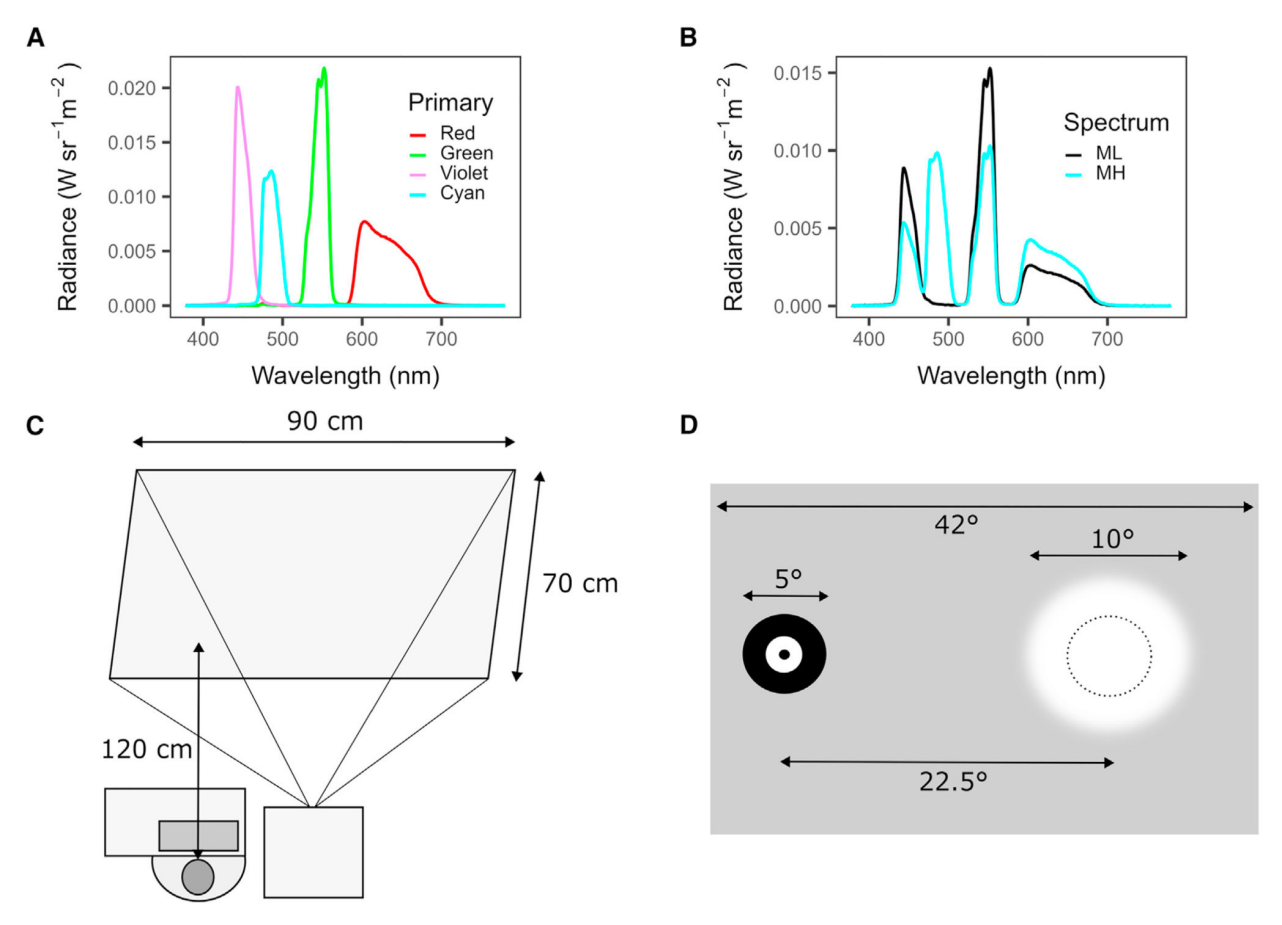
Multiprimary display and viewing geometry (A) Power spectra of the red, green, violet, and cyan primaries used to render the stimuli. (B) Power spectra of the standard observer melanopsin low (black) and melanopsin high (cyan) spectra. (C) Cartoon of the viewing geometry. The participant is seated at a desk next to the projectors, with their right eye closed. (D) Schematic of the paradigm when running flicker photometry/color-matching/chromatic contrast sensitivity experiments. Comprising a fixation point to left and an active stimulus (fuzzy spot) to the right. This schematic represents the 22.5° viewing condition; the dotted circle highlights the area where the fixation mark is presented when probing the 10° eccentricity.

A simple prediction of the hypothesis that melanopsin modulates color is that repeated switching between stimuli designed to provide equivalent excitation of cones (metamers), but differing in melanopsin excitation, should appear as a color flicker. Using a multiprimary (Figure 1A) projector system, we calculated melanopsin low (ML) and melanopsin high (MH) metamers, based upon the cone fundamentals of the CIE standard observer (Figure 1B), and applied minor adjustments to render them isoluminant for each of eight individuals by flicker photometry (STAR Methods; Figure S2). We then presented them as an annulus (central 5° blocked) covering the central 10° of visual space with sinusoidal modulations between ML and MH at 0.5 Hz.

**Figure 2:**
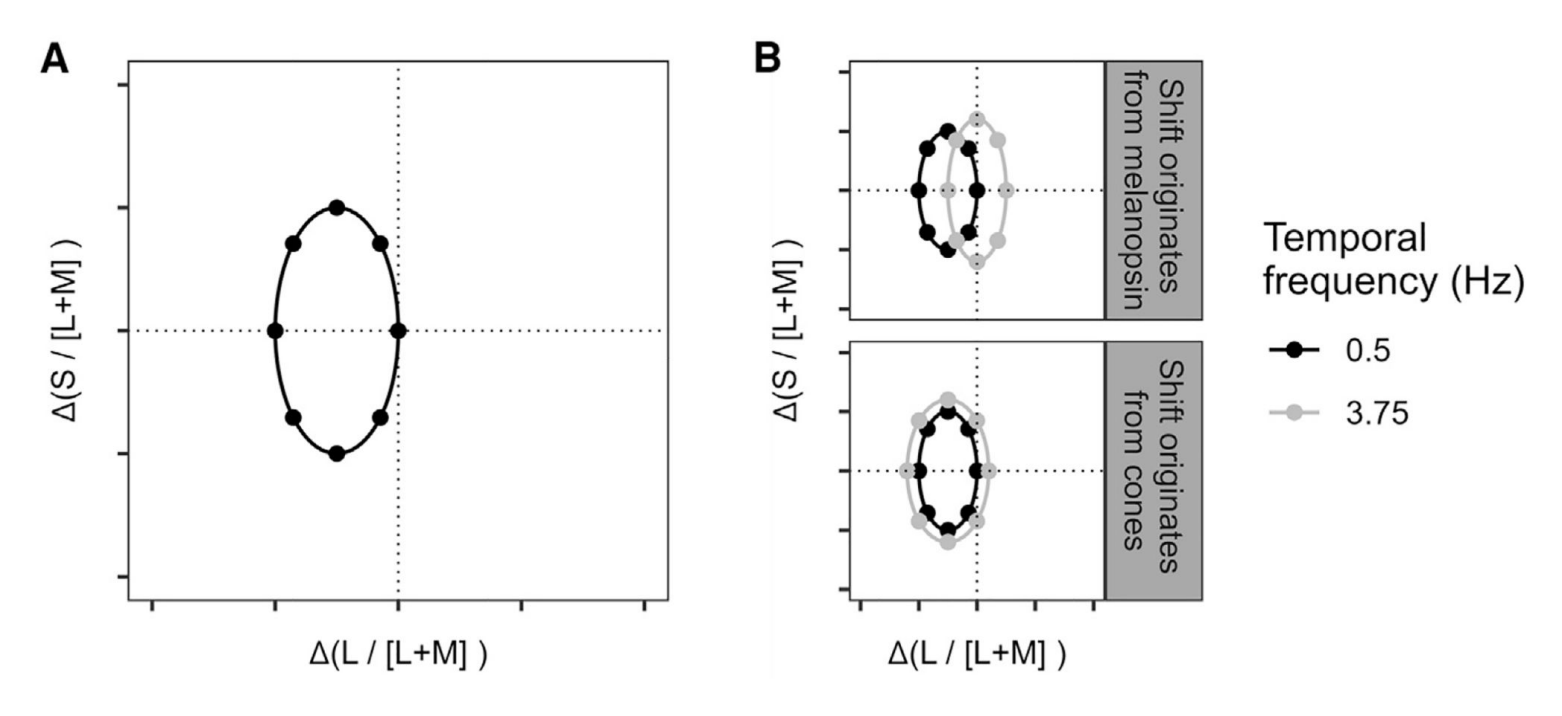
Predicted perceptual ellipses for MH in the presence or absence of a melanopsin contribution to color (A) Putative perceptual ellipse describing the range of MH spectra perceptually isochromatic with ML for a given individual. Dots represent the location in MacLeod-Boynton color space of eight MH spectra just detectable as a color flicker when interleaved with ML; dotted lines intersect at the predicted isochromatic coordinate for ML/MH, based upon the existing trichromatic color model. Here, the ellipse is shifted to the left with respect to the predicted isochromatic point, indicating that the color appearance of MH and ML cannot be normalized for this individual by applying the current cone-based color theory alone. One may conclude that either melanopsin contributes to color or that the cone spectral sensitivities of this individual simply differ from those used to construct the color space. (B) If the difference in color coordinates between the MH perceptual ellipse and the predicted isochromatic coordinate is produced by melanopsin (top), we expect its magnitude to be larger at 0.5 than at 3.75 Hz, given the known preference for low temporal frequencies of melanopsin. Conversely, if the deviation is unaffected by the temporal frequency at which it is assessed (bottom), this would indicate that it reflects inter-individual differences in cone spectral sensitivity.

Chromatic flicker was visible for 3 out of 8 observers. This outcome indicates that metamers designed according to the current trichromatic color model are frequently but not always perceptually isochromatic, leaving the potential contribution of melanopsin ambiguous. To provide a more quantitative estimate of any melanopsin effect, we ran a color-matching paradigm. Participants first adjusted the starting MH stimulus with isoluminant adjustments to the spectrum until no color flicker was apparent when interleaved with ML (Figure S3). They then added color to this revised MH stimulus in eight directions in MacLeod-Boynton color space until a flicker became just apparent (STAR Methods). This method produced eight different MH spectra per individual, which were at the limit of detectability versus ML. We planned then to map each of these eight spectra onto a conventional color space, in effect describing their predicted appearance according to the existing trichromatic model of color vision that does not incorporate melanopsin (Figure 2). It follows that if melanopsin makes no contribution to color, the centroid of the resulting ellipse should correspond to the color coordinate of an MH metamer designed according to existing color theory. Conversely, any melanopsin contribution to color will be apparent as a shift in the location of this centroid (Figure 2). However, an important feature of the color space calculation is that it assumes that for everyone, cone spectral sensitivity matches that of a nominal “standard observer.” In reality, small differences in cone spectral sensitivity from the standard observer are common thanks to variation in factors such as lens transmission and macular pigment density, which would similarly be apparent in a shift in location of the centroid. It follows that we need a method to determine whether any such shifts originate from melanopsin or simply divergence from the standard observer. To achieve this, we take advantage of melanopsin’s known preference for lower temporal frequencies by interleaving ML and MH at 0.5 and 3.75 Hz. As any color percept attributable to melanopsin should be more apparent at 0.5 Hz, we would expect a greater displacement of the perceptual ellipses at the lower temporal frequency (Figure 2B).

**Figure 3:**
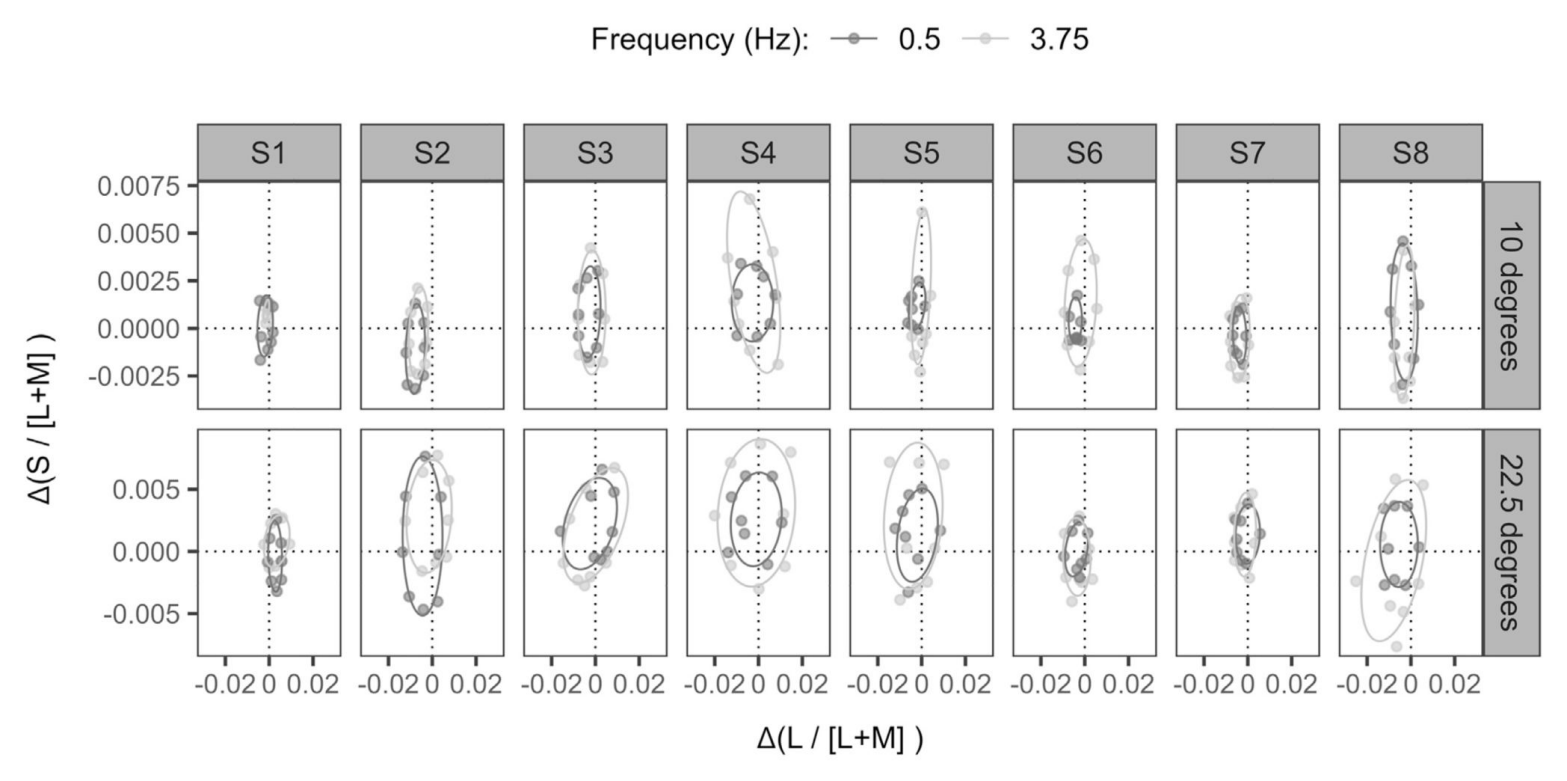
Perceptual ellipses for MH relative to the predicted MH/ML isochromatic point Perceptual ellipses (thin lines) describing the range of MH spectra perceptually isochromatic with ML for eight individuals, based upon the location in MacLeod-Boynton space of adjusted variants of MH required to produce a minimally detectable flicker (dots). Ellipses are close to the isochromatic coordinate predicted by the trichromatic color model (0,0) at both 0.5 Hz (black) and 3.75 Hz (gray) modulation frequencies and at both 10° (top) and 22.5° (bottom) eccentricities. Dotted lines drawn through predicted isochromatic point. For flicker photometry and color-matching procedures used for determining the isoluminant chromatic directions that were used per individual, see STAR Methods and Figures S2 and S3.

We decided to run this color-matching paradigm in both cen-tral 10° (reproducing conventional color-matching protocols) and peripheral 22.5° at which one source of inter-individual variation (macular pigment density) is minimized. We found that under all conditions, the perceptual ellipse around the color-matched MH was very close to the predicted isochromatic point (Figure 3). Centroids were displaced but only to a small extent, and in most cases, perceptual ellipses encompassed the predicted isochromatic point (0,0). These data therefore show that any melanopsin contribution to color is small and that for many individuals, MH and ML stimuli calculated based on the standard observer trichromatic color model will be perceptually metameric. This finding is also consistent with the difficulty many participants had in detecting an ML:MH flicker at the start of the experiment.

**Figure 4:**
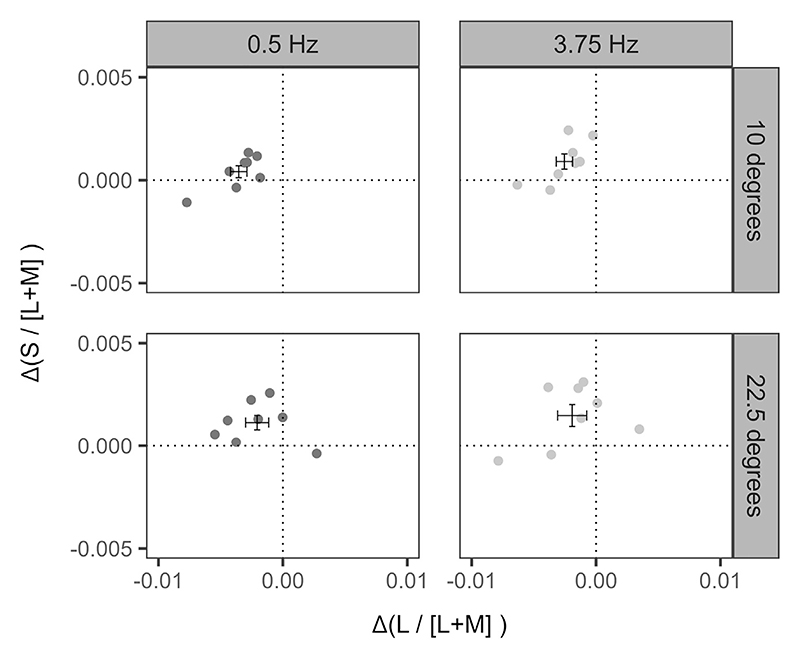
Outcome of MH/ML color-matching procedure is not altered by temporal frequency Centroids of MH perceptual ellipses (from Figure 3) plotted in MacLeod-Boynton color space relative to the predicted isochromatic point (0,0) for ML/MH, based upon current trichromatic color model. Datapoints show the centroid color coordinates for each of eight participants at 10° and 22.5° (top and bottom) and with MH interleaved with ML at 0.5 and 3.75 Hz (left and right). Bars show mean ± SEM across individuals; dotted lines drawn through predicted isochromatic point.

We applied a linear mixed-model analysis to explore the effect of eccentricity and temporal frequency on the centroids (Figure 4) of the perceptual ellipses (participant ID as random factor). The most important finding from this analysis is that there was no significant effect of temporal frequency at either eccentricity (p > 0.05). This observation argues that any deviation in MH centroid from the predicted ML/MH isochromatic point does not originate with melanopsin but rather with divergence of cone spectral sensitivity in our observers from that assumed in the CIE standard observer. The capacity for small inter-individual variations in pre-receptoral filtering to influence centroid locations in this paradigm is apparent from a comparison across eccentricities. Stimulus location did have a significant effect on MH centroid coordinates for the blue-yellow axis (t(23), p < 0.05), but this was within the range predicted by variations in macular pigment density (0.0085–0.022 based on previously reported^28^ estimates at an eccentricity of 3.75°, which was the center of the annulus in our 10° stimulus). Bayesian paired t tests showed moderate evidence against the null hypothesis that centroids are identical between temporal frequencies at 10° but in favor of it at 22.5° (10°, BF = 5.28 and 1.62; 22.5°, BF = 0.34 and 0.47 for L/(L + M) and S/(L + M) axes, respectively). In summary, these analyses reveal that while we cannot be certain that centroids are identical at the two temporal frequencies, any difference must be small. MH centroids are not significantly impacted by temporal frequency, and ellipses overlap almost entirely for all individuals. These findings indicate that melanopsin makes at most a very small contribution to any deviations in color of MH from that predicted by the current trichromatic model.

### Melanopsin enhances image persistence for positive and negative contrasts

**Figure 5:**
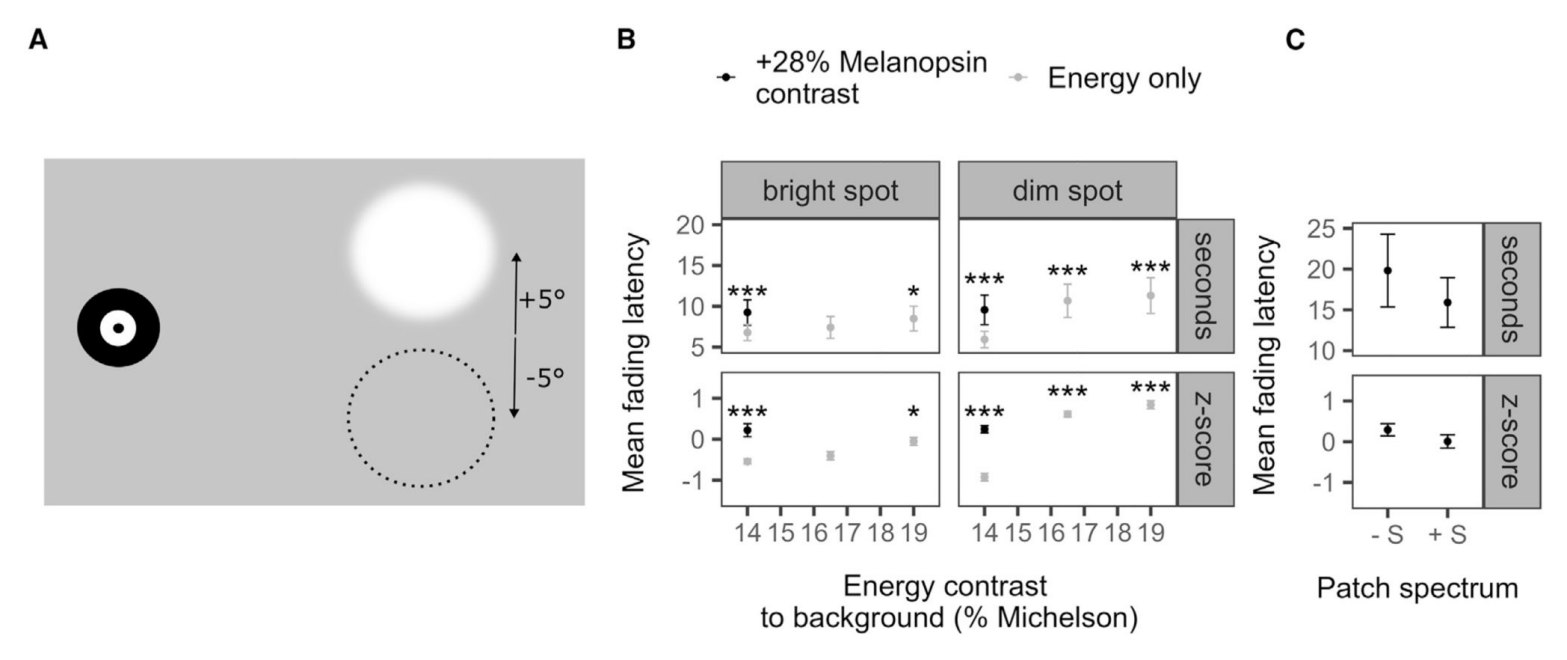
Troxler fading effects for melanopsin contrast but not color (A) Viewing geometry for the Troxler fading paradigms, which was identical to the viewing geometry of the color-matching and perceptual ellipse tasks, although in this task stimuli were presented only at 22.5° eccentricity and displaced vertically by 5° (fuzzy spot) or -5° (dotted circle) (alternated between trials). (B) Fading latencies for bright (left) or dim (right) spots in seconds (top) for stimuli varying in contrast. As the inter-individual variability in fading latency was large, *Z* scores were calculated by expressing fading latency as *Z* scores (bottom). Here, per participant, *Z*-transformed latencies were defined as ([latency – mean latency for that individual]/SD latency for that individual). To this end, we first calculated *Z* scores on a per-subject basis before averaging. Gray symbols comprise spectrally neutral (energy) differences in contrast (equivalent for all photoreceptors), black symbol a selective increase of 28% in melanopsin contrast super-imposed on the 14% energy spots. Datapoints show mean ± SEM for eight participants. Mixed linear models were fitted to the unstandardized data, separately for the bright and dim spot data, to reveal significant differences in latency from the 14% energy contrast. ***p < 0.001, **p < 0.01, *p < 0.05. (C) Troxler fading latencies in seconds (top) or standardized (*Z* score; bottom) for bright spots rendered in spectra corresponding to high melanopsin (MH, isochromatic 14% luminance + 42% melanopsin contrast), high melanopsin + negative S/(L + M) contrast (–S), or high melanopsin + positive S/(L + M) contrast (+S). Color offsets matched for S cone contrast. Data show mean ± SEM for eight subjects. A paired t test on the unstandardized data showed no significant differences between -S and +S (p > 0.05). See also Table S1 and Figure S4.

We next wished to test the hypothesis that just as neurons in the LGN show a sustained response to melanopsin-modulating light steps, so does human vision. Our approach is inspired by the Troxler fading phenomenon in which patterns fade into the background during fixation. To this end, we employed a paradigm, in which a blurred spot was presented at 22.5° eccentricity (Figure 5A), and the participant was tasked with indicating with a button press when the spot had disappeared. Four types of spots were used, three of which were spectrally neutral “energy” spots with 14%, 16.5%, or 19% Michelson contrast, compared with background for both luminance and melanopsin. The fourth was a spot with 14% luminance contrast and 42% melanopsin contrast (matched for luminance and color as above). The nominal contrasts of these stimuli are tabulated in Table S1. Participants completed 16 trials per condition, and the experiment was run twice, once using bright spots on the standard background and once using dim spots on an MH background. Six out of the original eight participants completed this experiment.

Our results (Figure 5B) show that the 19% (bright) and 16.5% (both dim and bright) energy spots faded slower than the 14% spectrally neutral reference, indicating that our paradigm was successful in capturing the effects of increasing stimulus contrast on fading latency. Importantly, this was also true for the dim and bright spots with additional melanopsin contrast, compared with the background. Thus, linear mixed-model testing showed that the bright 19% energy spots (t(21) = 2.785; p < 0.05) and both the dim 16.5% (t(21) = 4.43, p < 0.001) and 19% (t(21) = 6.17, p < 0.001) energy spots faded more slowly than the 14% energy references. This was also true for the bright (t(21) = 4.02, p < 0.001) and dim (t(21) = 4.16, p < 0.001) spots with additional melanopsin contrast.

The impact of melanopsin contrast on fading latency is consistent with our hypothesis that melanopsin enhances image persistence. However, we explored two alternative explanations for this finding: it reflects the known contribution of melanopsin to setting pupil size,^29,30^ and it indicates the possibility that the melanopsin modulation may impact cortical control of eye movements.^31^ To explore these possibilities, we analyzed data from the eye tracker that six out of eight participants wore during the Troxler fading tasks. Pupil diameter and gaze were analyzed for the task with bright spots. First, the mean pupil area did not significantly (p > 0.05) differ between conditions (pupil radius during the 42% melanopsin trials was on average 0.04 ± 0.09 mm smaller than during the 14% energy trials). It is therefore unlikely that the increased Troxler fading latency with increased melanopsin contrasts originates from differences in pupil diameter. This suggests that the stimuli were likely too small to significantly impact melanopsin-mediated pupil diameter. Second, all participants fixated equally well in both conditions. For the 14% energy condition, the mean ± SD of the 1.5 inter-quartile range (1.5 IQR) was 2.83° ± 1.09° horizontally and 2.45° ± 0.69° vertically. For the 42% melanopsin contrast spots, these 1.5 IQR values were 2.83° ± 0.99° and 2.09° ± 0.48°, respectively. This indicates gaze was almost never outside the fixation area but, more importantly, that there was no clear difference in fixation stability between the two conditions. Importantly, 2D fixation density landscapes constructed per participant per condition did not differ consistently between the two conditions (Figure S4). These additional control analyses therefore suggest that it is unlikely that fixation was better maintained in either of the two conditions or that pupil size differed, but it is most likely that the effects originate from melanopsin.

### Image persistence does not follow ipRGC (L + M)-on/S-off opponency

As our data suggest that ipRGCs mediate fading latency through their intrinsic light sensitivity channel (i.e., melanopsin), we finally asked whether the fading assay could reflect another reported property of ipRGCs: their spectral opponency. Our data above argue that this need not introduce a color percept, but it could in theory influence perceived brightness encoded by ipRGCs. Thus, if the Troxler fading effect were produced by ipRGCs with the described L + M-on/S-off opponency, then increasing S stimulation should antagonize the Troxler fading effect, while decreasing S should have the opposite effect. To this end, we presented isoluminant + isomelanopic chromatic versions (–S and +S) of the 42% melanopsin + 14% luminance patch (cone contrast presented in Table S1). We found that introducing S contrast in either direction delayed Troxler fading (Figure 5C), but a paired t test showed that there was no significant difference in the magnitude of this effect between +S and –S spots (t(7) = –1.54, p > 0.05). The Bayes factor following a Bayesian paired t test was 0.80, indicating anecdotal evidence in favor of the null hypothesis (no difference in latency between the two patches). Considering both analyses, we therefore fail to observe the predicted interaction between brightness (melanopsin + luminance) and color expected for the ipRGC spectral opponency.

## Discussion

In this work, we have addressed two outstanding questions regarding the role of ipRGCs in human vision, namely whether the spectral opponent mechanisms demonstrated in primate ipRGCs (L + M-on, S-off) have any perceptual correlate (e.g., a color percept), and whether melanopsin enhances perceptual persistence of static images as assessed via Troxler fading latency. Although we were not able to provide evidence for a role of ipRGCs in color vision, using a combination of color-matching and sensitivity contour quantification, our Troxler fading results do show that introducing melanopsin contrast enhances the persistence of images.

### Melanopsin enhances image persistence

The results of the Troxler fading experiment suggest involvement of melanopsin in image persistence: fuzzy spots presented in the periphery during fixation fade slower when their melanopic contrast to the background is high. This behavior fits well within the existing theoretical framework in which melanopsin is considered to encode low spatiotemporal features in the visual scene, with evidence coming from human psychophysics^26^ and electrophysiology studies.^20^ In this framework, ipRGCs compensate for the high spatiotemporal frequency bias of the luminance pathway.^32,33^ To understand the relevance of the Troxler fading experiment results with respect to this framework, it is useful to consider the conditions under which Troxler fading is most apparent in relation to the sensory properties of ipRGCs. Even during fixation, our gaze is never completely stable. Small eye movements continuously shift the locus of the image on the retina, such that different cones take turns in encoding parts of the visual scene.^34^ Therefore, when the local contrast surrounding the receptive field of a retinal ganglion cell is high, such as when the scene consists of high spatial frequency patterns, cones are repeatedly activated and relaxed, preventing adaptation and finally allowing for a stable representation of the image. This organization starts to break down, however, when eye movements are not able to produce repeated activation and relaxation of the cone cells. This may occur, for example, when eye movements are eliminated using stabilized retinal images^34^ or for low spatial frequency patterns for which the local contrast across the range covered by ongoing eye movements is low. In our Troxler fading paradigm, we effectively simulate the second scenario via fixation onto a scene with a blurred spot in the periphery. ipRGCs possess response characteristics that would allow them to reliably encode the spot in this situation. First, the melanopsin response is sustained during continuous light,^20^ allowing ipRGCs to reliably encode such static objects. Second, ipRGCs have large receptive fields^35^ and therefore are well equipped to encode low spatial frequency patterns, such as surfaces of objects where local contrast is low or objects with smooth edges. Together, these response properties would indeed predict the Troxler fading effect to be less apparent when a stimulus is rendered with a high melanopic contrast.

Perhaps the most direct experimental support for the prediction that Troxler fading latency should increase with higher melanopsin contrasts comes from electrophysiological recordings from the LGN in mice exposed to simulated active view of a visual scene, where changes in direction of view of varying magnitude were simulated by shifting the retinal locus of the image over time.^20^ In that experiment, melanopsin-responsive LGN cells were observed to be particularly involved in encoding coarse patterns during simulations of relative fixation. That work continued to present a quantitative model describing the unique contribution of melanopsin, which provides insight into its potential importance for everyday vision. According to that model, the degree to which melanopsin improves the dorsal LGN representation of scenes is negatively correlated with the frequency and magnitude of eye movements. However, the preponderance of low spatial frequency patterns in natural scenes means that the influence of melanopsin was apparent across changes in direction of gaze typical of many viewing scenarios beyond strict visual fixation.^20^ That work highlights the potential for the melanopsin contribution to image persistence revealed here to be relevant under a wide range of viewing conditions.

### No substantial melanopsin-mediated color percept

The currently accepted model of human color vision assumes three spectrally distinct inputs corresponding to the three classes of cone photoreceptors (the CIE standard observer cone fundamentals). In principle, these three cone fundamentals could already incorporate influence form a fourth photoreceptor (melanopsin) if it were engaged by the color-matching protocols under which they were derived. However, if that were so, we would expect the model to fail when probed with stimuli specifically designed to differ in effective intensity for this fourth receptor. In our case, the MH and ML stimuli should differ in perceived color even if matched for predicted color according to the cone fundamentals. Here, we actually test the converse of this prediction, namely that when matched for perceived color, MH and ML stimuli should differ in color as predicted by the current model. This approach enables us to use a color-matching paradigm to quantify any melanopsin effect rather than simply asking subjects whether MH and ML look alike. Thus, we define the version of MH that is perceptually equivalent to ML and then apply the cone fundamentals to determine its location in a standard color space (Figure 4). If the perceived color of our stimuli were accurately predicted by the existing color model, we expect the color-matched MH coordinates to be identical to ML (and those of the predicted MH metamer). In fact, for all observers the color-matched MH falls very close to that of ML in color space. Given the scale of the melanopsin contrast applied (30%), this finding alone suggests that any melanopsin contribution to color must be small. Nevertheless, color coordinates for perceptually matched MH and ML stimuli are not identical. We conclude that this small deviation originates with individual differences in cone spectral sensitivity from the fundamentals used to construct the color space rather than from a melanopsin contribution to color. Our argument relies on the fact that melanopsin is well known to prefer lower temporal frequencies,^16,20,26,27^ yet we detect no reliable shift in color coordinate between MH stimuli matched to ML under 0.5 versus 3.75 Hz modulation. In summary, while we cannot prove that melanopsin has absolutely no influence on color, under the circumstances employed here, its contribution must be sufficiently small not to have a material impact. The current trichromatic model for human color vision predicts perceived color exceptionally well even in the face of large melanopsin contrast.

Further evidence that color perception can be distinct from melanopsin comes from our Troxler fading work. We show that in a paradigm capable of revealing ipRGC influence, the impact of color does not follow that predicted by the reported chromatic opponency of ipRGCs. Thus, although blue light has been shown in some instances to inhibit ipRGCs,^1^ additional S cone contrast did not alter Troxler fading latency. This finding is consistent with the evidence from our color discrimination experiments that melanopsin and spectral opponent signals can be kept separate. In principle, that could be achieved by a single type of spectrally opponent ipRGC that was able to multiplex color and melanopsin information as two distinct outputs. Perhaps a more likely possible explanation, given the known diversity among the ipRGC population,^36,37^ is that the contribution of melanopsin to image persistence originates in a subset of ipRGCs that is not color opponent.

### Limitations of the study

One important caveat in our strategy for studying melanopsin in these experiments is that our metamers were not designed to be silent for rods. This constraint was necessary to obtain reasonable melanopic contrast, as the rod spectral sensitivity largely overlaps with that of melanopsin. Although the presence of rod contrast in our metamers may have influenced the color-matching and chromatic contrast sensitivity ellipse results, the contribution of rods to color vision is believed to be neglectable at the light intensities used here. Indeed, rods mainly contribute to color vision at very low light intensities,^38^ plateauing at light intensities as low as 100 photopic Td (our background light was 1,097 photopic Td, assuming a pupil area of 6.46 mm^2^ obtained with eye tracking). Rods, however, have also been shown to contribute to flicker detection at higher light intensities,^39^ which indicates that achromatic coding by rods extends well into the photopic range of light intensities under certain conditions. If rods then also contribute to the perceived contrast of the spot in the Troxler fading paradigm, the results could be explained by rod intrusions. However, these flicker detection paradigms rely on fast-changing stimuli that cause a phase-dependent interaction between rod and cone signals, which is unlike the nature of our sustained stimuli in the Troxler fading experiment. Indeed, in a study where individuals were asked to match the brightness of two spectra differing in rod activation, using the border-perception method,^40^ the rod contribution to brightness perception disappeared at adaptation levels as low as 100 scotopic Td (our background light was 1,910 scotopic Td, using an average pupil area of 6.46 mm^2^ obtained during eye tracking). In addition, the contrast sensitivity to single-rod-isolating flashes floors at between 300 and 1,000 scotopic Td.^41^ Therefore, it is unlikely that our findings are contaminated by rod intrusions.

A second limitation of our study is that the employed Troxler fading paradigm is not performance based and therefore strictly speaking not criterion-free. Indeed, the outcome parameter (fading latency) is subjective, and individuals may differ in their internal criteria of when fading occurred. In theory, this could lead to criterion bias between conditions that would contribute to our findings. We think that this is most unlikely for the ML versus MH comparison. Participants had no a priori expectation of the outcome of this experiment, and the melanopsin-modulating spots were indistinguishable from one another (when participants were asked if they could see a flicker between these spectra at the start of the perceptual ellipse task, they all responded negative, which was in fact a prerequisite to continue that task). Moreover, despite the substantial inter-individual differences in fading latency, the MH stimulus faded slower for every individual. Criterion bias is harder to exclude for the +S and −S patches, which clearly look different, and the outcome of that part of the study should be interpreted with that in mind.

## Conclusions

In summary, although we failed to find evidence in support of a melanopsin-mediated color percept, we do provide evidence that Troxler fading latency is reduced by increasing melanopsin contrast. This highlights the role of melanopsin in encoding low spatiotemporal frequency information in everyday visual scenes, which is most clearly observed when demonstrating the visibility of low spatial frequency patterns under visual fixation. From a functional perspective, our fading and color discrimination data are consistent with a model in which melanopsin is more important for brightness than color percepts. Indeed, luminance, with its high-frequency bias, is a more obvious candidate to be complemented by the low-frequency tuning of melanopsin than color channels, which themselves already have good low spatiotemporal frequency sensitivity.^42,43^ It therefore appears that the contribution of melanopsin to human vision is most clearly demonstrated when using stimuli to which the luminance channel is least sensitive.

## Star⋆Methods

### Key Resources Table

**Table:** 

REAGENT or RESOURCE	SOURCE	IDENTIFIER
Software and algorithms		
MATLAB	The Mathworks	https://www.mathworks.com/products/matlab.html
Psychtoolbox	Psychtoolbox	http://psychtoolbox.org/
R	The R Project for Statistical Computing	https://www.r-project.org/
Pupil Capture	Pupil Labs	https://docs.pupil-labs.com/

### Resource Availability

#### Lead contact

Further information and requests for resources, reagents, or raw data should be directed to and will be fulfilled by the Lead Contact, Tom Woelders (tom.woelders@manchester.ac.uk)

#### Materials availability

This study did not generate new unique reagents.

### Experimental Model and Study Participant Details

#### Humans

Eight participants (white European, 1 graduate student, 7 full-time employees) were recruited for this study (5 male, 3 female) with an average age of 36 years (range 22-57). All participants participated in all experiments, whereas 6 individuals (1 female, 5 male; age range 22-57) additionally underwent eye tracking in the Troxler fading experiments. All participants demonstrated normal color vision, as assessed by the Ishihara color blindness test. Due to relatively small sample size and small variation, analysis of the influence (or association) of sex, gender, ancestry, race and ethnicity, socioeconomic status or a combination of these factors could not be performed. This may limit the generalization of the results if any outcome measures are correlated with these variables.

#### Ethics

Ethical approval was obtained from the University of Manchester Ethics commission (approval number #2023-16374-27669). Informed consent was obtained from all participants.

### Method Details

#### Light stimulator and spectra

All stimuli were created using an in-house engineered multiprojector system.^26^ In short, the spatially aligned outputs of two projectors (one containing red, green, and violet primaries and the other containing a cyan primary) were projected onto a Lambertian surface to allow for a four-primary control per pixel (Figure 1A). Spectra were calculated using the cone and melanopsin fundamentals of the CIE 2006:170-1^44^ standard 10 degree observer. All participants adapted to a grey background (Macleod Boynton L / (L+M) and S / (L+M) coordinates of 0.68 and 0.023 respectively, 210 cd m^-2^, 0.13 W sr^-2^ m^-2^ melanopic radiant flux, 1097 photopic Td, 1910 scotopic Td, assuming a pupil area of 6.46 mm^2^ obtained with eye tracking) before performing the flicker photometry, color matching, perceptual ellipse and Troxler fading (bright spots) tasks. This background light intensity was chosen to saturate the rods (which we do not control for in our metamers) to move out of the mesopic range where rods have been reported to provide small contributions to color perception,^38^ limiting rod intrusion for the color discrimination paradigms. The melanopsin low reference stimulus used in flicker photometry, color matching and perceptual ellipse tasks was a bright version of the background (280 cd m^-2^, same color coordinates). The default melanopsin high stimulus was a nominal metamer of the ML stimulus at 30% Michelson contrast for melanopsin. Therefore, during these tasks the stimulus area was designed only to modulate melanopsin. In the Troxler fading task with dim spots, the MH metamer was used as the background (280 cd m^-2^), which is the only task where a different background was used. Spectral measurements were obtained using a spectroradiometer (SpectroCAL MKII; Cambridge Research Systems, UK).

#### Projector calibration and validation

To obtain a linear relationship between primary intensity settings and primary radiance, the projectors were gamma corrected. First, the primaries were measured at 15 regular intervals (from min to max). Then, 5^th^ order polynomials were fitted to the data and the fits were used to construct a color lookup table (CLUT). This CLUT was then used in the Pyschtoolbox OpenGL pipeline (see viewing geometry and stimuli below). To test whether linearization of the primaries was successful (i.e., the predicted and measured intensity values are matched), we measured 256 spectra ranging from ML to MH and compared the measured contrasts with respect to ML to the predicted contrasts. The standard deviation of the residual Michelson contrast was 0.44% for melanopsin and 0.38% Michelson contrast for luminance, demonstrating acceptable linearization. We furthermore explored the temporal characteristics of the projectors to ensure proper temporal alignment between the two projectors as well as exclude potential artifacts due to the sluggish nature of LCD technology. A detailed description of this validation procedure is presented in the supplemental information (Figure S1). In short, the temporal limitations of the LCD display are not expected to play a role if temporal frequencies < 7.5 Hz are used.

#### Age and macular pigment corrections

When calculating the MacLeod Boynton chromaticity coordinates presented in this paper, we applied the CIE 2012:203^45^ age-dependent lens correction. For the stimuli presented at the 22.5 degrees eccentricity, we adjusted the photoreceptor fundamentals by assuming a decrease of macular pigment density of 0.095^46^ compared to those used for the 10 degrees viewing condition, to account for the fact that there is virtually no macular pigment expected to be present in the far periphery. This means that whenever we refer to the standard observer trichromatic model for human color vision, we refer to the individual age and macular pigment (for the 22.5 degrees eccentricity) corrected versions of the CIE 2006:170-1^44^ standard observer model.

#### Viewing geometry and stimuli

Stimuli were rendered using the OpenGL pipeline (and custom GLSL shaders) provided by the MATLAB (v R2021B) Psychtoolbox (v 3.0.18) library. Psychtoolbox was set to output video in dual display mode ensuring the displays were temporally aligned. All participants were seated in front of the lower left corner of the screen at a 120 cm distance (Figure 1C). The width and height of the screen were 93 and 70 cm respectively, spanning a viewing angle of 42 degrees horizontally and 32.5 degrees vertically. At all times, participants were asked to fixate at a small dot in the center of a black circle (2.5-degree radius, to block light hitting the macula) with their right eye closed. In the experiments where the stimuli were presented at the 22.5 degrees eccentricity, the fixation mark was presented 22.5 degrees to the left of the center of the stimulus, whereas when the 10 degrees field of view was probed, the fixation mark was presented at the center of the stimulus, creating an annulus. The stimuli used were spots with a radius of 5 degrees (plus a gaussian-tapered edge of ~3 degrees) presented at a vertical offset of 0 degrees (flicker photometry, color matching and sensitivity contour paradigms, Figure 1D) or -5 or 5 degrees (Troxler fading experiments, Figure 5A). We opted for stimuli with smooth edges to 1) minimize effects of chromatic aberrations, which are expected to mainly play a role at higher spatial frequencies and 2) to enhance the Troxler fading effect.

#### Flicker photometry

Flicker photometry was employed to find the participant-specific isoluminant direction vectors in primary space for the melanopsin and the approximate L / (L+M) and S / (L+M) chromatic axes (i.e., standard observer axes corrected for luminance bias). This procedure was therefore run three times (once per probed direction) for both eccentricities (10 degrees and 22.5 degrees). To find each isoluminant direction we presented a spot with a sinusoidal modulation in spectrum (e.g., from ML to MH for the melanopsin direction) at a frequency of 7.5 Hz. The participant added luminance (along the standard observer isomelanopic luminance direction) to the MH spectrum until no apparent flicker was present, for four trials, of which the average was calculated to serve as the starting spectrum for the next eight trials. These were used to find the added or subtracted luminance at which the flicker reappeared, resulting in a total of eight trials per probed direction (four per upper/lower sensitivity boundary), from which the average was taken to calculate the closest estimate of the isoluminant + isomelanopic direction (Figure S2). As the projector system is driven by an LCD display, which have a poor temporal resolution especially at high contrasts, we decided to reduce the contrast of the melanopic spectral pair to 7% for this paradigm (5.81% effectively, see Figure S1), and the final MH spectra with 30% melanopsin contrast to ML were derived from these results by extrapolation. For the melanopsin version, isoluminant centers were close to zero, and the standard observer prediction well within the detection boundaries for most participants, indicating no clear deviations from the standard observer predicted isoluminant point.

#### Color matching

MH stimuli were color matched to ML to provide a starting point for constructing perceptual ellipses. Participants were presented with a spot with a sinusoidal modulation between ML and MH, for all four combinations of the two eccentricities (10 degrees and 22.5 degrees) and temporal frequencies (0.46875 Hz and 3.75 Hz). These temporal frequencies were chosen to avoid temporal artifacts as they are harmonics of the 60 Hz refresh rate of the projectors. Participants were instructed to match the MH color to that of the ML reference, by navigating the isoluminant + isomelanopic chromatic plane obtained from the flicker photometry calibration. For each condition, participants completed 8 trials starting from different color offsets equally spaced across the isoluminant + isomelanopic plane around MH (Figure S3), from which a perceptual metamer was calculated as the average over these 8 trials.

#### Chromatic contrast sensitivity contours

This experiment was performed once for all combinations of eccentricity and temporal frequency. At the start of the procedure, participants were asked whether a flicker was present. No participants reported a visible flicker, indicating that the spectra were truly metameric (both cone-mediated luminance and color). The procedure itself was similar to the color matching paradigm with the exception that here, each trial started with ML alternating with the visually metameric MH spectrum obtained from the color matching experiment results. Participants were requested to adjust the chromatic contrast (by adjusting their matched version of MH) to the point where a chromatic flicker was just about visible, along each of eight isomelanopic + isoluminant color directions obtained from their individual flicker photometry results. At the end of the procedure, ellipses were fitted to the chromaticity coordinates of the thresholds, and the ellipse centroids were taken as the most accurate and final estimates of the melanopic metamers.

#### Troxler fading

The Troxler fading procedure was only employed for the 22.5 degrees eccentricity. A trial consisted of making a bright spot appear at 22.5 degrees eccentricity by pressing the space bar and then pressing the space bar again when the spot had faded (this also removed the spot), yielding a fading latency. To prevent possible aftereffects that might occur when presenting the stimulus at the same position at each trial, the stimulus was presented at a vertical offset of -5 and 5 degrees for odd and even numbered trials respectively (Figure 5A). The background was the same as for all other tasks. Four bright spots with different melanopsin and luminance contrasts compared to the background were then probed: three spectrally neutral spots at a Michelson contrast (for both luminance and melanopsin) of 14, 17 and 19 percent, and one spot that had a 14 percent (nominal) luminance contrast but also a 42 percent (nominal) melanopsin contrast compared to the background. Note that for the latter spot, the luminance contrast compared to the background was the same as the dimmest spectrally neutral spot, but its melanopic contrast was three times higher. We also employed an inverted version of the Troxler fading procedure, by setting the background to MH and presenting dim spots at negative energy contrasts of 14, 17 and 19 percent (for both luminance and melanopsin), and one with a negative luminance contrast of 14% combined with a negative melanopsin contrast of 42%. An overview of the contrasts employed can be found in Table S1. Each spot was presented 16 times, resulting in a total of 128 trials (both bright and dim spots).

#### Troxler fading with chromatic patches

To test whether Troxler fading shows the spectral opponency we expect from ipRGC electrophysiology (L+M on, S off), we repeated the Troxler fading paradigm with two different patches, which included 2 isoluminant isomelanopic chromatic versions of the 42% melanopsin patch (18.46% nominal Weber contrast in either the – S / (L+M) or + S / (L+M) chromatic directions), using the S / (L+M) chromatic direction vector obtained from the flicker photometry protocol. The cone contrasts of these patches were identical but in reverse polarity. An overview of the contrasts employed can be found in Table S1.

#### Eye tracking and pupillometry

During the Troxler fading task (bright spots), participants wore an eye tracker (Pupil Labs, Pupil Core, 30 Hz sampling frequency) to explore whether reported effects could be originating from differences in fixation stability or pupil diameter. To assess the location of gaze, we used the surface tracking capability of the Pupil Capture software, such that gaze positions were expressed in normalized screen coordinates (range: 0 to 1). These values were converted to degrees of visual angle, from which deviations from the mean were calculated for data collected during the 14% energy and 42% melanopsin trials. We then constructed 2D density landscapes of the gaze coordinates per participant per condition (Figure S4A), as well as calculated 1.5 x interquartile ranges to assess deviations on the horizontal and vertical axes (see results). We also constructed difference landscapes from the normalized (unity peak) 2D landscapes to assess systematic differences in fixation between both conditions and found no indication of systematic fixation differences between conditions (Figure S4B). We also calculated the average pupil diameter for both conditions (again, only data during stimulus presentation analyzed) and found that the pupil diameters in both conditions were almost identical (see results).

### Quantification and Statistical Analysis

The statistical details (test-statistics, p-values, degrees of freedom, particular tests used) can be found in the text. In the figures, asterisks are used to highlight significance levels. Details of n for each experiment (n = number of participants) can be found within Figure legends. A p-value of 0.05 was used to define significance. Data throughout the manuscript are presented as mean ± SEM, unless explicitly stated otherwise. All statistical analyses were performed in R (v4.1.2) using the RStudio shell (v 2021.09.1). Linear mixed models were fitted using the lme4 (v1.1-31) package, and significance values were extracted from the fits using the lmerTest (v3.1-3) package. Linear mixed effects models were fitted for the chromaticity coordinates of the ellipse centroids to test for effects of viewing condition or temporal frequency (fixed effects), while controlling for interindividual variation (participant ID as random effect). Linear mixed models were fitted on the Troxler fading latencies as well, one for the bright and one for the dark spots, with the four spot types as the categorical fixed factor and participant ID as random effect. To further analyze negative results, Bayesian paired t-testing was performed using the BayesFactor (v0.9.12-4.4) package in R (ttestBF function), on the ellipse centroids and the fading latencies with colored stimuli. The 2D density landscapes of fixation were constructed using the kde2 (kernel density estimation) function from the R MASS (v7.3-60) package.

## Supplementary Material

Supplemental information can be found online at https://doi.org/10.1016/j.cub.2023.10.039.

Supplemental Information

## Data Availability

All data reported in this paper will be shared by the lead contact upon request. This paper does not report original code. Any additional information required to reanalyze the data reported in this paper is available from the lead contact upon request.

## Highlights

- Melanopsin enhances image persistence for low spatiotemporal frequency patterns
- The image persistence originates from melanopsin rather than ipRGC spectral opponency
- Melanopsin provides no substantial color percept

## In brief

Woelders et al. demonstrate that melanopsin enhances image persistence, especially for low spatiotemporal details. Their color-matching experiments, using high melanopsin contrast metamers at frequencies both within and surpassing melanopsin’s temporal sensitivity range, reveal that melanopsin does not provide a substantial color percept.
