# Supplementary material for: Melanopsin enhances image persistence
Source: Curr Biol. Author manuscript; Available in PMC 2026 Aug 6. (PMC7619323; doi:10.1016/j.cub.2023.10.039)
Supplement: Supplementary file 1 [file EMS216119-supplement-Supplemental_Information.pdf]

Current Biology, Volume *33*

Supplemental Information

Melanopsin enhances image persistence

Tom Woelders, Annette E. Allen, and Robert J. Lucas

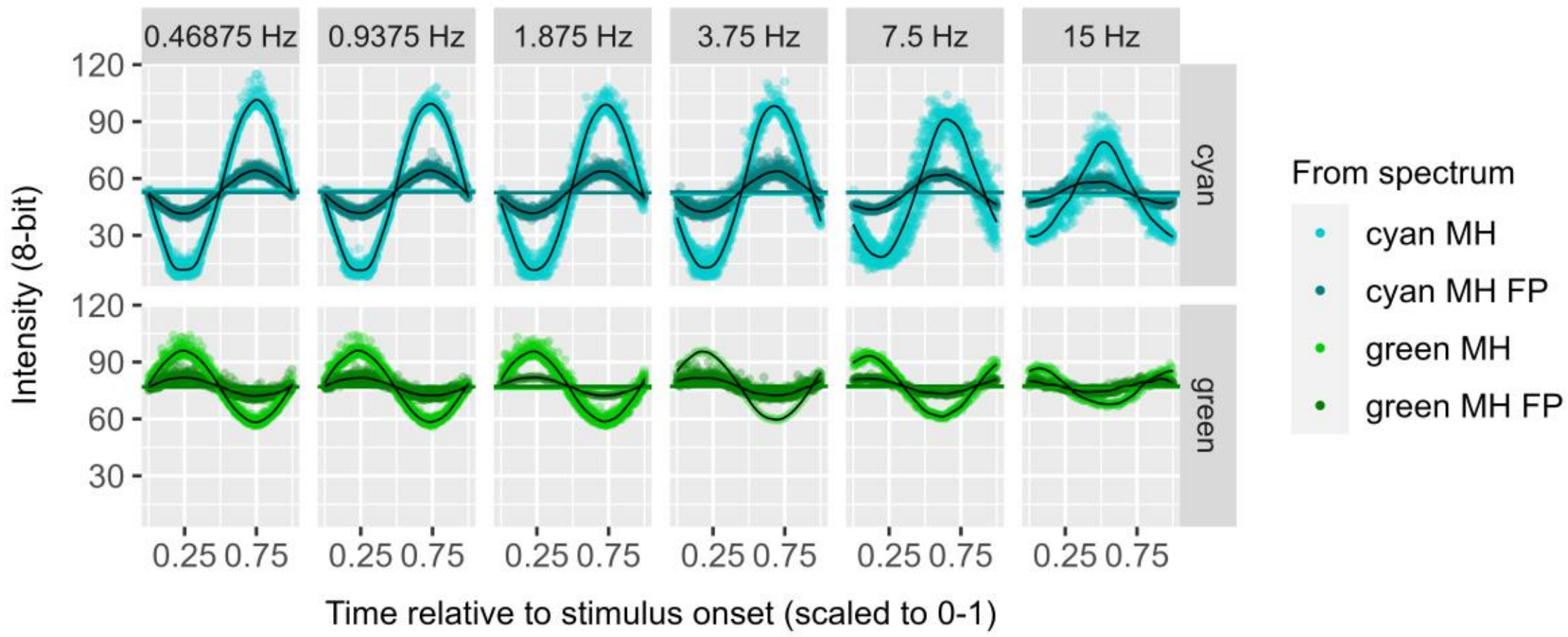

Figure S1. Temporal limits of the multiprimary projector system, related to STAR methods. As LCD technology is known to perform poorly at high temporal frequencies, and especially at large contrasts, we performed a photodiode validation (200 Hz sampling frequency) experiment to explore the (temporal) limitations of the system. Presented are peristimulus traces (recorded using a photodiode) for two primaries (cyan and green) in response to sinusoidal modulations at a range of frequencies (harmonics of the 60Hz projector refresh rate). Smooth loess (locally weighted scatterplot smoothing, span: 1/4) fits are drawn in black through the dots for each of the four conditions, together with horizontal-colored lines showing the averages over time. The chosen intensities and amplitudes were based on the stimuli used in the flicker photometry (FP) protocol (green FP and cyan FP, bright colored dots, which is a ML-MH modulation at a 7% melanopsin contrast) and the standard observer ML-MH modulation (green MH and cyan MH, dark colored dots). When the standard observer ML-MH modulation is applied, the amplitude of the intensity modulation is significantly reduced at the two highest frequencies tested (7.5 and 15 Hz). Furthermore, temporal alignment between the two primaries (each coming from a separate projector) is violated. However, temporal alignment was achieved for the FP modulation at 7.5 Hz. We then explored whether the amplitude reduction that is observed between the 7.5 Hz and slower conditions was proportionally identical for the green and the cyan primaries. This was indeed the case (reduction of 18.6% and 16.7% for cyan and green primaries respectively, corresponding to a difference between primary settings of 0.4 units on an 8-bit scale). This means that the relative intensities of the primaries are not altered by this amplitude effect. Additionally, this amplitude reduction at 7.5 Hz was symmetrical (averages do not change). These two results together indicate that the only practical implication of this amplitude reduction has is that the applied melanopsin contrast is about 17% lower than expected (5.81% instead of 7% Michelson contrast). This validation procedure therefore shows that although amplitude was reduced at 7.5 Hz for the primaries, the ML-MH FP modulation remained on the nominal melanopsin axis, justifying the extrapolation of the 30% MH spectra from the flicker photometry results.

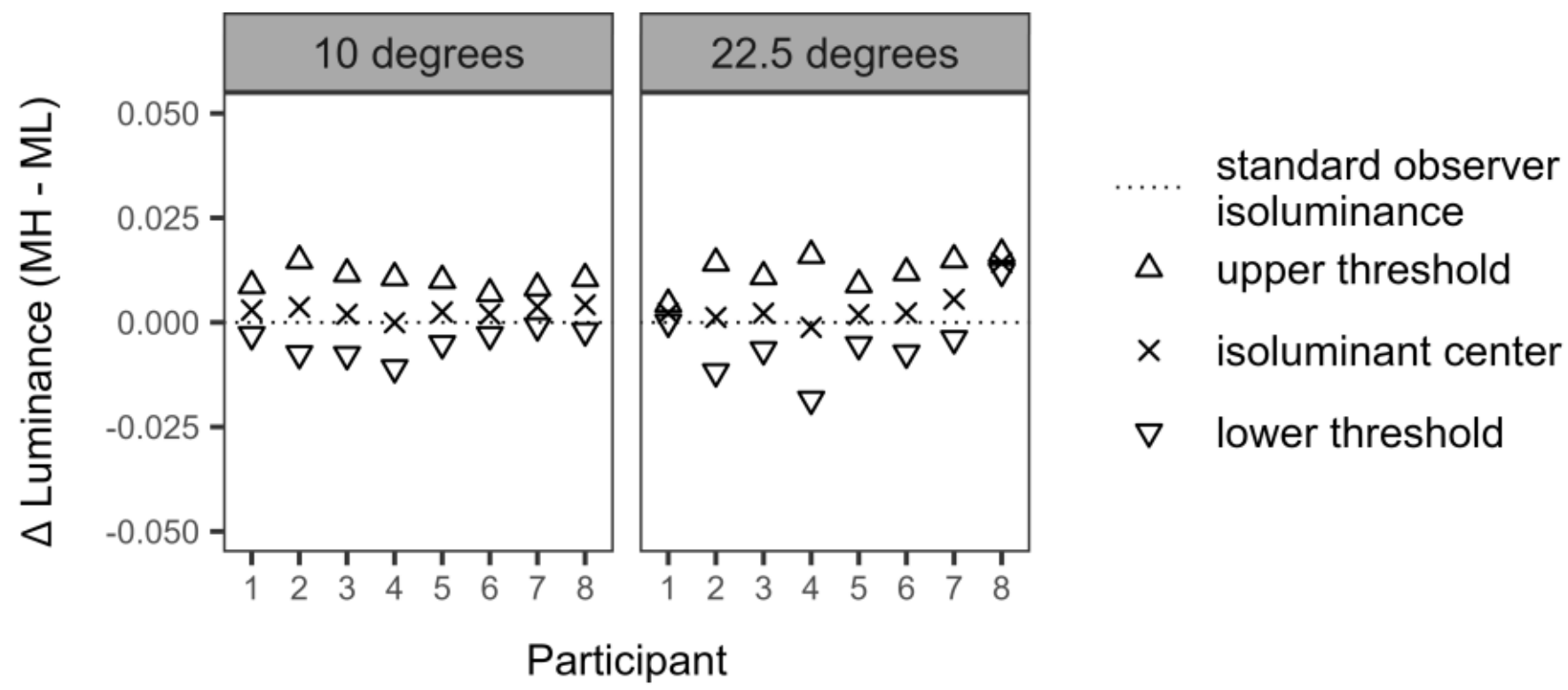

Figure S2. Flicker photometry results for melanopic isoluminant direction, related to Figure 3 and STAR methods. Luminance offsets applied to the standard observer MH spectrum by each participant (S1-S8) to reveal a detectable flicker when interleaved with ML at 7.5 Hz at 2 eccentricities. Upper and lower sensitivity thresholds per subject are depicted with upward and downward pointing triangles respectively. Cross shows isoluminant point for each participant and eccentricity, calculated as the midpoint between the upper and lower detection thresholds. The dotted horizontal line indicates the isoluminance as predicted for the standard observer (offset=0). Similar experiments (results not shown) were performed for the two chromatic axes to construct isoluminant isomelanopic chromatic vectors in primary space.

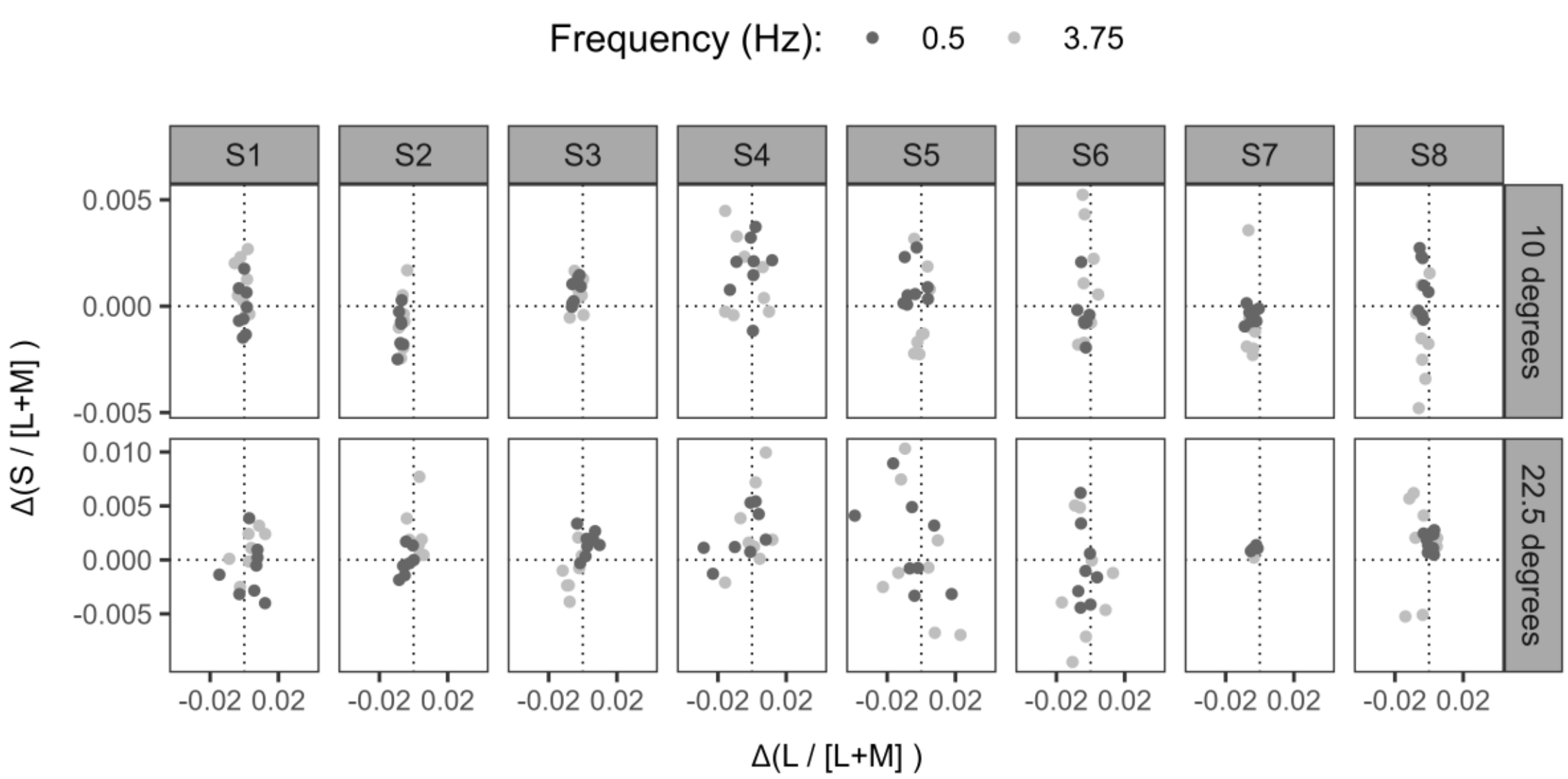

Figure S3. Color coordinates of MH spectra color-matched to the ML reference, related to Figure 3 and STAR methods. Chromaticity coordinates of MH spectra following adjustment to match apparent color of ML at each of two temporal frequencies and eccentricities in each participant (S1-S8). Coordinates are presented in Macleod Boynton space as the difference from the isochromatic coordinates predicted for ML/MH according to the standard observer trichromatic color model (0, 0). Each panel shows the outcome of 8 iterations of the color matching at each temporal frequency (0.5 and 3.75Hz; black and grey symbols respectively) covering a range of starting with color offsets. Dotted lines drawn through the predicted isochromatic coordinate for the standard observer for reference.

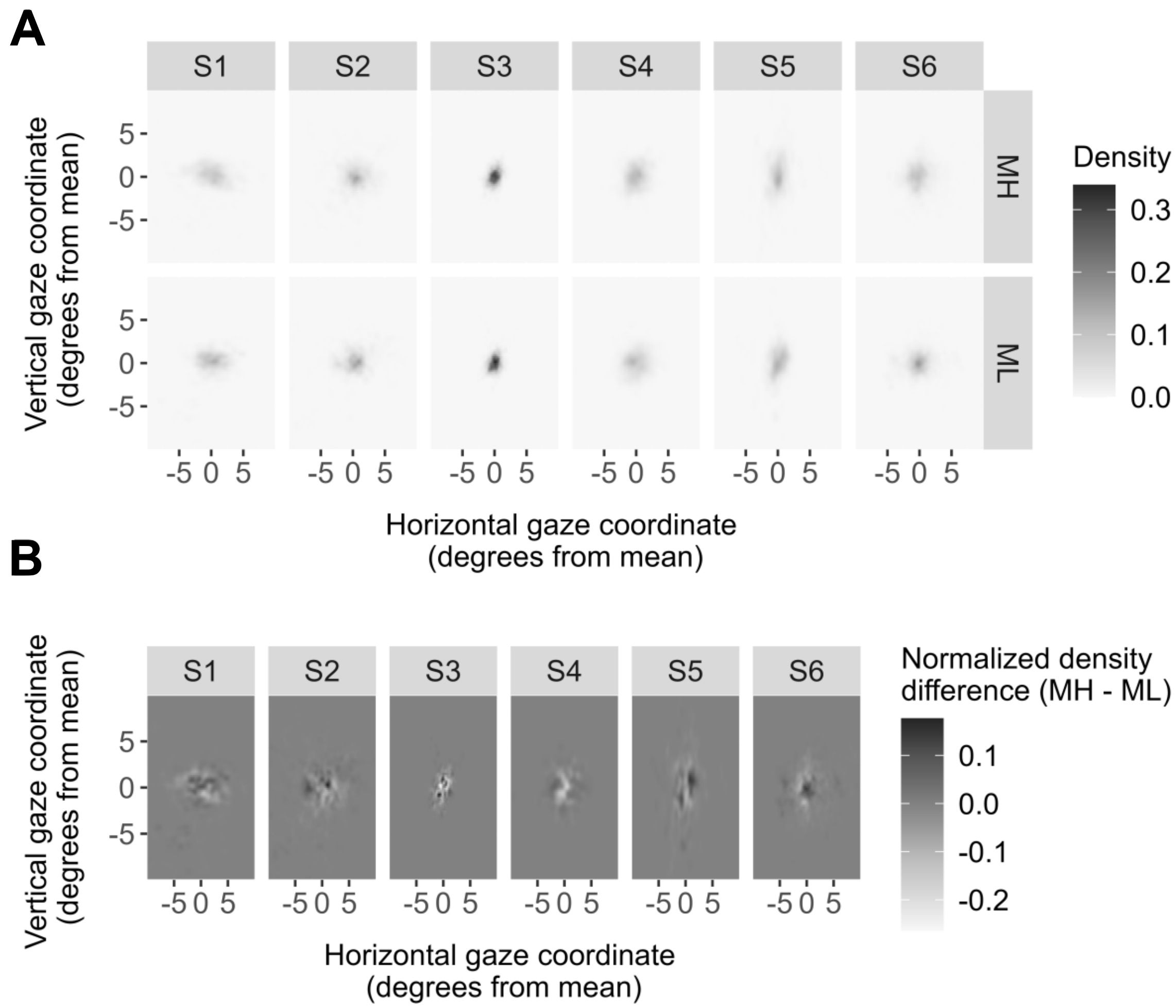

Figure S4. No differences in fixation stability between the low and high melanopsin contrast conditions (bright spots version), related to Figure 5 and STAR methods. A) To assess any systematic differences in fixation between the ML (14% energy contrast) and MH (14% luminance, 42% melanopsin contrast) conditions in the bright spot version of the Troxler fading task, 2D density plots are presented for the gaze data for six participants who underwent the Troxler fading paradigm wearing an eye tracker. For ML, the mean ± sd 1.5 inter-quartile range (1.5 IQR) was 3.58 ± 1.38 degrees horizontally and 3.19 ± 0.90 degrees vertically. For MH these 1.5 IQR values were 3.58 ± 1.25 degrees and 2.72 ± 0.62 degrees respectively. These data show that all participants reliably fixated within the 5-degrees fixation area. **B)** We additionally constructed difference 2D landscapes (MH – ML) between normalized (unity peak) versions of these 2D landscapes and found no systematic differences in fixation between the ML and MH conditions. If fixation had been more stable in the ML condition compared to MH (leading to faster Troxler fading in the ML condition as an alternative explanation), these difference landscapes should consistently show light areas with a dark surround (i.e., more stable fixation in the ML condition), which is not what the data show.

Paradigm	Stimulus	Reference	Predicted Weber contrast (%) S cone	Predicted Weber contrast (%) M cone	Predicted Weber contrast (%) L cone	Predicted Michelson contrast (%) Luminance	Predicted Michelson contrast (%) Melanopsin
Flicker photometry	Mel high (MH)	Mel low (ML)	0.00	0.00	0.00	0.00	7.08
	+ (L / [L+M])	- (L / [L+M])	0.00	-5.18	2.65	0.00	0.00
	+ (S / [L+M])	- (S / [L+M])	31.17	0.00	0.00	0.00	0.00
Color matching (after match) and chromatic contrast ellipse (at trial start)	Mel high (MH)	Mel low (ML)	0.00	0.00	0.00	0.00	30.00
Troxler fading (bright spots)	Background	Mel low (ML)	-31.58	-32.66	-32.62	-14.03	-13.73
	14% energy + 28% melanopsin	Background	31.58	32.66	32.62	14.03	42.00
	14% energy	Background	37.89	39.19	39.15	14.03	13.73
	16.5% energy	Background	44.21	45.72	45.67	16.40	16.40
	19% energy	Background	31.58	32.66	32.62	18.60	18.60
Troxler fading (dim spots)	Background	Mel high (MH)	0.00	0.00	0.00	0.00	0.00
	14% energy + 28% melanopsin	Background	-24.00	-24.62	-24.60	-14.03	-42.00
	14% energy	Background	-24.00	-24.62	-24.60	-14.03	-14.03
	16.5% energy	Background	-27.60	-28.31	-28.29	-16.48	-16.48
	19% energy	Background	-31.20	-32.00	-31.98	-19.04	-19.04
Troxler fading (colored spots)	- (S / [L+M])	Mel high (MH)	-18.46	0.00	0.00	0.00	0.00
Troxler fading (colored spots)	+ (S / [L+M])	Mel high (MH)	18.46	0.00	0.00	0.00	0.00

Table S1. Predicted cone, luminance and melanopsin contrasts for all stimuli used in the experiment, related to Figure 5 and STAR methods. Cone contrast is expressed in Weber contrast (calculated as (stimulus – reference) / reference) and both luminance and melanopsin contrasts are expressed in Michelson contrast (calculated as [stimulus – reference] / [stimulus + reference]). All contrasts are calculated for the CIE 2006 standard observer.
